# Uncomplicated pharyngitis due to family transmission of hypervirulent *Klebsiella pneumoniae*

**DOI:** 10.1007/s10096-025-05043-6

**Published:** 2025-01-23

**Authors:** Hannah K. Gray, Sanchi Malhotra, Shekina Gonzalez-Ferrer, Gregory D. Whitehill, Alisa C. Chalmers, Shangxin Yang

**Affiliations:** 1 Department of Pathology and Laboratory Medicine, David Geffen School of Medicine, University of California, Los Angeles, CA USA; 2 Division of Pediatric Infectious Diseases, Department of Pediatrics, David Geffen School of Medicine, University of California, Los Angeles, CA USA; 3 Division of Infectious Diseases, David Geffen School of Medicine, University of California, Los Angeles, CA USA; 4 Department of Medicine, David Geffen School of Medicine, University of California, Los Angeles, CA USA; 5 UCLA Clinical Microbiology Laboratory, 11633 San Vicente Blvd, Los Angeles, CA 90049 USA

**Keywords:** Hypervirulent *Klebsiella pneumoniae*, Pharyngitis, Family transmission, Genomic surveillance, Genotyping

## Abstract

We describe two cases of uncomplicated pharyngitis caused by hypervirulent *Klebsiella pneumoniae* (hvKp) in a family, initially in an immunocompetent adolescent, followed by possible household spread resulting in similar presentations in the patient’s parent. Genomic analysis confirmed hvKp from the two cases were genetically identical and typed as K2-ST3252. Nasopharyngeal carriage and respiratory secretion/droplet may play an important yet underrecognized role in the transmission of hvKp. Enhancing routine screening for hvKp in the upper respiratory culture, followed by genotyping provides an effective pathway for early diagnosis.

## Introduction

Pharyngitis is a common pediatric complaint, most commonly due to viral etiology. Bacterial pharyngitis is most often caused by Group A *Streptococcus*, and less commonly by *Arcanobacterium haemolyticum*, other streptococci, *or Fusobacterium*. Rarely, atypical bacteria, fungi, or sexually transmitted infections may also be implicated [1].

Hypervirulent *Klebsiella pneumoniae* (hvKp), generally characterized by hypermucoviscosity and higher virulence than classical *Klebsiella pneumoniae* (cKp), causes invasive and difficult-to-treat disease in immunocompetent hosts. Typically, hvKp infections cause splenic and hepatic abscesses, bacteremia, pneumonia, endophthalmitis, or central nervous system (CNS) infection [2]. Non-invasive isolation of hvKP has also been described such as urinary tract infection, wound infection or asymptomatic rectal carriage [3–5].

Six distinct capsular types have been identified, and multilocus sequence types (MLST) 23 and 86 are most frequently reported. At our institution, K1-ST23 was predominant, and abscesses were the most common presentation [2]. Virulence factors associated with hypervirulence and hypermucoviscosity are carried on plasmids, including genes encoding increased capsule production and iron acquisition [6]. Hypermucoviscosity, as indicated by string test > 5 mm is a sensitive albeit nonspecific predictor of hypervirulence, and thus genotyping is critical to identify hvKp. Markers with strong predictive association for hvKp include: *iuc*A (aerobactin siderophore), *iro*B (salmochelin siderophore), *peg*-344 (putative transporter), and *rmp*A and *rmp*A2 (regulators of capsule production) [7]. Following a passive surveillance study for hvKp which showed ~ 3% of hvKp are found in clinical non-urine isolates, our institution implemented routine genotyping of clinical Kp isolates that are string test positive by whole-genome sequencing (2).

## Materials and methods

The throat culture was performed using a set of 4 culture media including sheep blood agar, selective Strep agar, chocolate agar and MacConkey agar. The inoculated plates were incubated at 35^o^C with 6–10% CO2 for 48 h. Bacterial species was identified by MALDI-TOF (BioMerieux VITEK MS v3.2). Antibiotic susceptibility test was performed by broth microdilution following the Clinical and Laboratory Standards Institute (CLSI) guidelines. String test and whole-genome sequencing methods were described previously (2). Single nucleotide polymorphism (SNP) and antimicrobial resistance (AMR) gene analysis was performed using CLC Genomics workbench (Qiagen) and ResFinder Database v1.2 [8]. Gene mutation analysis was performed using Geneious Prime (Dotmatics). Visualization of plasmids mapping and comparison was generated by Proksee (https://proksee.ca/) [9]. Parental and patient consent was obtained.

## Results

A thirteen-year-old previously healthy female presented to urgent care with complaint of one day of sore throat with associated congestion, fatigue and subjective fever, in the absence of cough, conjunctivitis, lymphadenitis, vomiting or abdominal pain. The patient had no travel history, sick contacts, or recent hospitalization, and no notable past medical or surgical history. She exhibited an erythematous posterior oropharynx without exudates or cervical lymphadenopathy, and normal respiratory, cardiovascular, abdominal and skin exams.

Point-of-care group A *Streptococcus* antigen and influenza PCR tests were negative. Throat swab was submitted for bacterial culture and nasopharyngeal swab for SARS-CoV-2, influenza, and RSV PCR. The viral PCRs were negative. Further viral testing was not sent on this visit as it would not have changed management at the time. Bacterial culture grew predominant amount of *Klebsiella pneumoniae*. Antibiotic susceptibility testing (AST) was performed, and the isolate was universally susceptible to all tested drugs (Table 1). The visibly mucoid isolate was string-test positive. As per institutional protocol, the suspected hvkp isolate was further genotyped by an in-house whole genome sequencing (WGS) based test to detect virulence factors associated with hypervirulence [2].

**Table 1:** Antibiotic susceptibility results

Antibiotic	MIC	Interpretation†
Piperacillin/Tazobactam	≤ 8	Susceptible
Cefazolin	1 or 2*	Susceptible
Ceftriaxone	≤ 1	Susceptible
Ceftazidime	≤ 0.5	Susceptible
Ceftolozane/Tazobactam	≤ 0.5	Susceptible
Cefepime	≤ 0.5	Susceptible
Meropenem	≤ 0.25	Susceptible
Imipenem	≤ 1	Susceptible
Ertapenem	≤ 0.25	Susceptible
Amoxicillin/Clavulanate	≤ 2	Susceptible
Gentamicin	≤ 1	Susceptible
Tobramycin	≤ 1	Susceptible
Amikacin	≤ 4	Susceptible
Ceftazidime/Avibactam	≤ 2	Susceptible
Ciprofloxacin	≤ 0.25	Susceptible
Levofloxacin	≤ 0.5	Susceptible
Trimethoprim/Sulfamethoxazole	≤ 1/20	Susceptible

*One isolate tested cefazolin MIC = 1 and the other tested MIC = 2; results were within a normal variation of 2-fold dilutions and both were interpreted as susceptible

†Per CLSI M100 breakpoints

**Fig. 1:**
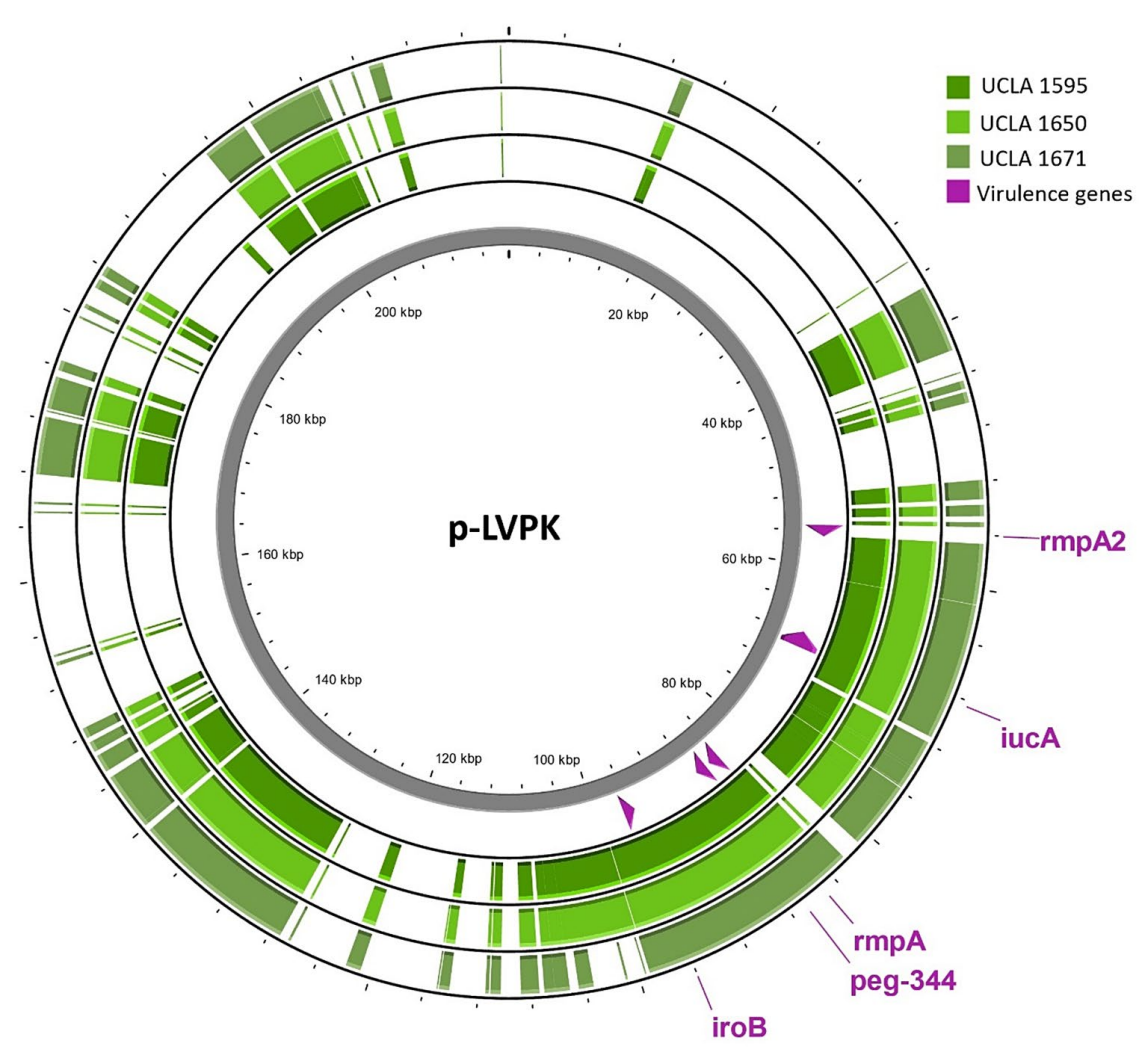
Alignment of the p-LVPK sequence to three pharyngitis isolates (UCLA 1595, 1650, and 1671)

The isolate (Isolate1) carried four full-length genetic markers (*iucA*,* iroB*,* peg-344*, and *rmpA*), and was typed as capsule type K2 and sequence type (ST) 3252 by using Pathogenwatch web application (https://pathogen.watchaccessed9/13/2024). Two plasmids, typed IncFIA(HI1) and IncFIB(K), respectively, were detected, with the IncFIA(HI1)-type plasmid closely related (~ 98% pairwise identity) to a well-characterized hvKp plasmid FO834905 carrying virulence genes *iuc*A, *iro*B, *rmp*A, and *peg-344*, annotated as *pag-O* [10]. Four virulence genes were successfully aligned with the canonical hvKp plasmid p-LVPK (NC_005249) with a high pairwise identity (*rmpA* = 99.1%, *peg-344* = 99.7%, *iroB* = 99.2%, *iucA* = 99.0%), but none of the isolates (isolate 1, 2, or 3) showed sequence alignments with *rmpA2* (Fig. 1). Protein alignment of the virulence biomarkers with those of the p-LVPK plasmid showed slight differences in the amino acid sequences ranging from 2 to 6 mutations (Table 2). Moreover, two resistance genes, *OqxA* and *OqxB* - conferring resistance to fluoroquinolone, were also found. Interestingly, the phenotypic AST did not show fluoroquinolone resistance; the reason for the discrepancy between genotype and phenotype remains to be investigated. Notably, the isolate is closely related to a K2-ST66 type clinical isolate (B055) identified in Australia, 2020 (NCBI Reference: NZ_CP072200.1).

**Table 2:** Protein pairwise identity and amino acid changes of virulence biomarkers upon alignment with the canonical hvKp plamid p-LVPK

	Virulence gene				
	*iucA*	*iroB*	*peg-344*	*rmpA*	*rmpA2*
Protein Pairwise ID (%)	99.5	99.5	98.7	96.7	Not detected
Mutation					
				F36L	
				V44I	
			Q46P	L80I	
	T9A		I112F	R132Q	
	Q37E	L230M	S121L	I133V	
	P370L	P245S	A208D	K142Q	

Virulence biomarkers *iucA*,* iroB*,* peg-344*, and *rmpA* were aligned to the canonical p-LVPK. All three isolates displayed the same pairwise identity and amino acid changes

The patient was prescribed amoxicillin/clavulanate upon culture result but did not take the regimen as she already felt improved. Five weeks later, the patient re-presented to her primary care physician (PCP) with three days of congestion, sore throat, and fatigue. The patient’s mother was also seen by this PCP and the patient’s brother by a separate PCP, both with sore throat at the same time. Throat cultures were collected from the patient and her mother (Table 3). SARS-CoV-2, influenza, and RSV PCR were repeated at re-presentation and again negative.

**Table 3:** Clinical history and microbiologic results

Timepoint (month)	0	1	2
*Symptoms*			
Index	Present	Present	NS
Mother	NS	Present	Present
Sibling	NS	Present	NS
*Throat Culture*			
Index	hvKP (Isolate1)	LPGNR*	n/a
Mother	n/a	hvKP (Isolate2)	hvKP (Isolate3)
Sibling	n/a	n/a	n/a
*Treatment*			
Index	NT	AMC	n/a
Mother	n/a	AMC	NT
Sibling	n/a	AMC	n/a

Abbreviations: NS, no symptoms; hvKP, hypervirulent Klebsiella Pneumoniae; n/a, not-applicable (either throat culture not collected, or treatment not considered due to lack of symptoms); AMC, amoxicillin-clavulanate; NT, no treatment; LPGNR, lactose positive gram-negative rods. *The isolate was morphologically identical to the previous hvKp isolate but was not further worked up due to the assumption that it was the same hvKp

The patient’s throat culture again grew lactose positive gram-negative rods but was not further speciated as her PCP declined the further workup based on the assumption that it was the same hvKp. The culture from the patient’s mother, five weeks after the primary patient’s specimen was collected, also yielded a *K. pneumoniae* isolate, which was string test positive and confirmed to be hvKp by WGS. The isolate (Isolate2) was susceptible to all tested antibiotics. Both isolates were typed as K2-ST3252, and phylogenetic analysis of the core genome revealed a difference of four single nucleotide polymorphisms (SNPs), indicating the two isolates were closely related. The brother’s PCP did not obtain a throat culture.

All three family members were prescribed and completed 10 days of amoxicillin/clavulanate. One month later, the patient’s mother presented for a routine physical exam. She requested a throat culture due to three days of a scratchy throat. The throat culture was again positive for hvKp despite a completed antibiotic course one month prior. The isolate (Isolate3) was sequenced, and genomic analysis showed it was genetically identical to the mother’s first throat culture isolate (Isolate2), with only two SNPs difference, and equivalent plasmid and virulence gene profile (Fig. 1). The mother did not return for follow-up so no further treatment was given. The other family members did not have recurrence of sore throat. No one developed any invasive infections.

## Discussion

*K. pneumoniae* frequently colonizes mucosal epithelium of the nasopharynx and gut, with reported colonization rates of 5–35% in Western countries [6]. Despite mucosal colonization, dissemination of cKp is rare in immunocompetent hosts without underlying conditions. As a result, *K. pneumoniae* isolated in upper respiratory cultures is generally considered normal flora, and further workup is not routinely performed. Uncomplicated pharyngitis is an atypical presentation for hvKp, and few cases of similar infections have been reported. A previously described case in a 33-year-old German male was associated with unprotected oral sex practices and was characterized by painful tonsillopharyngitis with visible uvular ulcers [11]. The only organism identified was a hvKp typed as K2-ST66, and the patient was treated with octenidine mouth and throat rinse. In another case reported from Taiwan, a patient presented with pharyngitis mimicking malignancy, and *K. pneumoniae* was isolated from the throat culture and determined to be the cause [12]. However, the isolate was not further investigated for hvKp, despite a high likelihood due to the endemicity of hvKp in Taiwan.

To our knowledge, our study is the first case report of presumed hvKp pharyngitis with strong evidence of family transmission. Alternate viral etiologies were not fully excluded, which is a limitation of our report. However, the absence of cough, rhinorrhea, and conjunctivitis and the recurrent episodes of pharyngitis with genetically identical strains still strongly supports hvKp as the source.

As previously described, hvKp infections are likely underdiagnosed and underreported in the United States [2]. While cases are largely community acquired, the modes, extent, and epidemiology of transmission is not well established. In this case, household transmission is highly probable, resulting in an atypical presentation of hvKp confirmed in two family members. The primary patient was not initially adherent to prescribed antibiotic treatment, which may have provided an opportunity for household spread. Our patients did not have any co-morbidities or risk factors for developing invasive disease. Nasopharyngeal carriage and respiratory secretion/droplet may play an important yet underrecognized role in the transmission of hvKp. Enhancing routine screening for hvKp in the upper respiratory culture, followed with genotyping provide an effective and pragmatic pathway for early diagnosis. The definition of hvKp as truly hypervirulent is constantly evolving, as the proposed diagnostic testing modality has shifted from string test evaluation to genetic analysis of 4 out 5 hypervirulent genes. However, the presence of only 4 out of 5 virulence genes, regardless of the specific biomarkers involved, may lead to an overestimation of hvKp [13]. Using murine models, Russo et al. demonstrated that the genotype of 5 out of 5 hypervirulent genes detected predicts hvKp with 96% accuracy, while the predictive value dropped to only 84% with 4 out of 5 genes detected [13].The major limitation of this study is the lack of confirmation of hypervirulence by murine models, which represents the gold standard. Due to the same reason, we also could not assess the impact of the limited number of mutations found in the four virulence genes. Nonetheless, recognizing and initiating prompt investigation for hvKp pharyngitis may allow early evaluation for signs and symptoms of invasive disease or prevent dissemination in higher risk patients, especially as community rates increase.

## Data Availability

The datasets generated and/or analyzed during the current study are available in the GenBank BioProject PRJNA1179472 under the accession numbers: SAMN44494244, SAMN44494245, and SAMN44494246.
